# Supplementary figures and images for: Elevated neutrophil-to-lymphocyte ratio and predominance of intrahepatic cholangiocarcinoma prediction of poor hepatectomy outcomes in patients with combined hepatocellular–cholangiocarcinoma
Source: PLoS One. 2020 Dec 11;15(12):e0240791. doi: 10.1371/journal.pone.0240791 (PMC7732129; doi:10.1371/journal.pone.0240791)

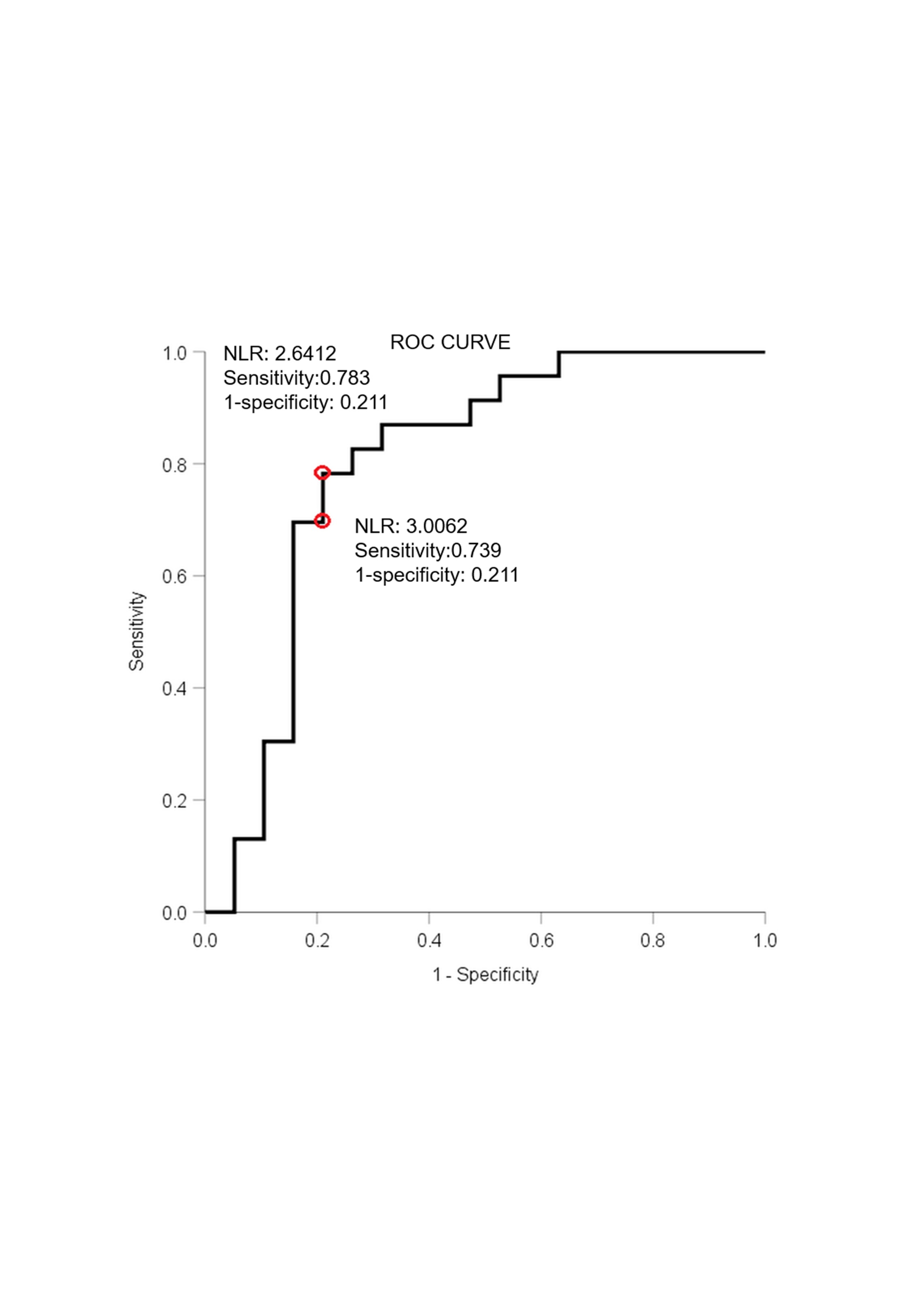

Supplement: S1 Fig — Threshold values were determined using the ROC curves, and the value with the highest sensitivity and specificity was calculated. (TIF) [file pone.0240791.s001.tif]
